# Epidermoid cancers of the oropharnyx

**DOI:** 10.1054/bjoc.2000.1761

**Published:** 2001-05

**Authors:** J L Renaud-Salis, M P Blanc-Vincent, J Brugère, F Demard, A Faucher, G Gory-Delabaere, J Pinsolle

**Affiliations:** Institut Bergonié, Bordeaux; Cancer Research Campaign, Paris; Institut Curie, Paris; Centre Antoine Lacassagne, Nice; Centre Val d'Aurelle Paul-Lamarque, Montpellier; Hôpital Pellegrin Tripode, Bordeaux


*British Journal of Cancer* (2001) **84**(Supplement 2), 37–41
© 2001 FNCLCC
doi: 10.1054/ bjoc.2001.1761, available online at http://www.idealibrary.com on IDEAL®

### Epidermoid cancers of the oropharynx

**JL Renaud-Salis[1], MP Blanc-Vincent[1,2], J Brugère[3], F Demard[4], A Faucher[1], G Gory-Delabaere[2,5] and J Pinsolle[6]**

[1]Institut Bergonié, Bordeaux; [2]FNCLCC, Paris; [3]Institut Curie, Paris; [4]Centre Antoine Lacassagne, Nice; [5]Centre Val d'Aurelle Paul-Lamarque, Montpellier; [6]Hôpital Pellegrin Tripode, Bordeaux

There are about 7000 new cases of epidermoid carcinoma of the oropharynx per year making up 95% of malignant oropharyngeal tumours. The incidence has been steadily rising and France has the highest incidence of this cancer in the world.

The vast majority of oropharyngeal cancers are squamous cell carcinomas. This document does not consider other rare neoplasms (e.g. mucosal melanoma, plasmacytoma, soft-tissue sarcoma or minor salivary gland tumours) occasionally found in the head and neck. The management of patients with oropharyngeal cancer requires a multidisciplinary team of individuals with expertise in all aspects of the special care needs of these patients.

These guidelines were validated in June 1999 by the working group. An update is planned for 2001/2

### INITIAL ASSESSMENT

The initial 'work-up' of a patient with oropharyngeal cancer involves clinical examination coupled with imaging studies.

The clinical examination must assess the patient's performance status and any signs suggestive of probable extensive disease (e.g. trismus, reduced lingual protraction, earache) (standard). The history taking must cover alcohol and tobacco use and quality of life issues (standard). A general anaesthetic may be necessary for the assessment of locoregional extension and for tumours at the base of the tongue. The tumour must be measured. The morphology of the tumour (e.g. whether it is exophytic, infiltrating or ulcerative), should be noted along with any infiltration of adjacent structures (e.g. the mandible) or of muscles (masticators, muscles at the base of the tongue).

Initial assessment includes a biopsy for histological confirmation. Clinical examination of cervical lymph node areas must note the presence of nodes, their sites, dimensions, mobility and number (standard).

Standard investigations are a chest X-ray (CXR) to look for synchronous bronchial tumours and orthopantomography to detect any dental defects that should be corrected prior to treatment.

Optional examinations include:

- oesophagoscopy (to look for synchronous tumours)
- CT scan or MRI of the head and neck (in case of suspicion of deep muscle and/or bone involvement)
- cervical ultrasonography (to evaluate the extension of cervical nodes in obese patients with no palpable lymphadenopathy)
- panendoscopy (if there is a history of prolonged alcohol and tobacco use)
- bronchoscopy (if there is suspicion of a second cancer on CXR).

A search for metastases is only indicated if there are clinical symptoms and signs suggestive of disease spread.

### CLASSIFICATION

The TNM classification of the International Union Against Cancer (UICC) is the one most commonly used.

### PROGNOSTIC FACTORS

Prognosis is related to:

- the degree of locoregional extent as assessed clinically (the size and mobility of the primary tumour, extension to muscle or bone, the presence of lymph nodes and whether they are fixed)
- histological factors linked to the tumour (tumour grade, thickness, quality of the surgical margins)
- histological factors linked to lymph nodes (invasion, capsular rupture, nodal site and a number of involved nodes).

The role of tumour markers as prognostic factors is currently being evaluated. Stage at diagnosis is the factor most predictive of survival. In general, the survival rate of patients with locally advanced disease (stage III or IV) is less than half that of patients with early stage disease (stage I or II). Distant metastases are uncommon at presentation.

### TREATMENT MODALITIES

The therapeutic techniques include surgery, radiotherapy, brachytherapy and combined radiotherapy and chemotherapy. As there are no randomized trials to guide management in oropharyngeal cancer, all therapeutic decisions should be made by a multidisciplinary team, in order to define the treatment best suited to each individual case.

### Tumours of the base of the tongue

There is no difference between external radiotherapy, radiotherapy plus brachytherapy or surgery with or without radiotherapy for local control of T1–T3 disease that is in the order of 70–90% (level of evidence C). For T4 tumours, the rate of local control is considerably lower. There may be an advantage in favour of combination surgery and radiotherapy.

### Tumours of the tonsillar fossae and anterior pillars

For limited stage disease (T1–T2), external radiotherapy, radiotherapy plus brachytherapy and surgery followed by postoperative radiotherapy give equivalent results in terms of local control (90% for T1 and 75–80% for T2 tumours) (level of evidence C). For T3 tumours, the combination of radiotherapy and brachytherapy is better (65–72%) than radiotherapy alone (37–67%) (level of evidence C). Surgical series do not detail results in terms of T stage. The results of surgery alone are not directly comparable to those of radiotherapy/brachytherapy but are similar. For T4 tumours, no comparison between different treatments is possible. The failure rate is greater than that for T3 tumours (level of evidence C).

### Tumours of the soft palate and uvula

The three treatment modalities (surgery, radiotherapy, radiotherapy and brachytherapy) give equivalent rates of local control for limited stage disease (70–100% for T1 and 60% for T2 tumours) (level of evidence C). There is no consensus as to the best modality for stage T3/T4 disease.

### Lymph node areas

The results of treatment of cervical lymph node areas with surgery or radiotherapy are equivalent for N0 and N1 disease with a high rates of control (96–100% for N0, 90–93% for N1 disease). If nodes are involved, postoperative radiotherapy seems to reduce the frequency of recurrence (level of evidence C). There is no consensus as to the relative efficacy of radiotherapy and surgery for T3 disease, but as the rate of local recurrence tumours is high (in the order of 30%), if either method is used alone. They are usually combined. This applies to the treatment of lymph node areas for all the cancers of the upper aerodigestive tract.

### CHEMOTHERAPY

Neoadjuvant and adjuvant chemotherapy do not improve locoregional control or survival in oropharyngeal cancer (level of evidence A). Combined radiochemotherapy, either alone or in addition to surgery, can improve both local control and survival in extensive but potentially curable lesions of the oropharnynx (T3, T4a, N0 to N3) when compared to surgery and radiotherapy (level of evidence A). The role of radiochemotherapy as compared to radiotherapy alone (particularly with hyperfractionation), remains to be confirmed in clinical trials.

Neoadjuvant or adjuvant chemotherapy should not be offered to patients with cancer of the oropharynx who are potentially treatable by locoregional methods (level of evidence A). Combination radiochemotherapy given postoperatively for cancers at risk of local recurrence, or given as sole treatment for extensive cancers, are options. If possible, these patients should be included in clinical trials.

### TREATMENT STRATEGY

### T1, N0, M0 tumours of the oropharnyx

There is no standard. Surgery and radiotherapy have equivalent efficacy (level of evidence B). Simple surgical excision by the oral route, brachytherapy or external radiotherapy are therapeutic options (Figure 1).

The choice of treatment depends on the likelihood of functional and cosmetic sequelae, on social considerations and the views of the patient. Surgery is preferable for lateral lesions if it can be done via the oral route, as this will result in very few functional sequelae and in young patients lessens the risk of second malignancies. When the margins of surgical excision are narrow (less than 5 mm) or invaded, additional radiotherapy is recommended (level of evidence B).

Elective treatment of lymph node areas is optional. If the primary tumour is treated surgically, this should consist of an exploration of the supra-omohyoid area, followed by a selective neck dissection if one or more nodes are positive, preserving the sternocleidomastoid muscle, jugular vein and spinal accessory nerve. For lateral tumours, cervical irradiation can be limited to the ipsilateral cervical zones without compromising local control (level of evidence B). Treatment of local recurrence gives the same results in terms of cervical control and survival (level of evidence B). The choice of treatment of lymph node areas should be made according to the preference of the patient and the multidisciplinary team.

### T1, N1, M0/T2, N0–N1, M0 tumours

There is no standard. Surgical excision plus exploration of the supra-omohyoid nodes (with clearance if the nodes are positive), external radiotherapy to the tumour and the cervical nodes or conventional radiotherapy plus brachytherapy are the therapeutic options. The choice of treatment is individualized and dependent on performance status, age and patient preference.

The therapeutic options for the primary tumour include surgery and external radiotherapy or brachytherapy plus external radiotherapy, the efficacy of which are equivalent for this type of lesion with a local control rate in the order of 90% (level of evidence B). Surgery is preferable for lateral tumours and infiltrating or ulcerative tumours which are likely to respond less favourably to radiotherapy. Additional radiotherapy is necessary when the surgical margins are narrow (less than 5 mm), or involved, to reduce the risk of local recurrence (level of evidence B). Radiotherapy alone, or radiotherapy plus brachytherapy, is preferable for those in whom surgery is likely to produce a considerable functional deficit.

Elective treatment of uninvolved lymph node areas (N0) can be considered for larger tumours (T2) in order to reduce the risk of cervical relapse (level of evidence B). For lateral tumours, cervical irradiation can be limited to ipsilateral cervical nodes (level of evidence B). In patients who have had surgery, the presence of unequivocal nodal disease, histological involvement of several nodes or capsular rupture, are indications for postoperative irradiation to reduce the risk of cervical recurrence (level of evidence B).

### T3, N0–N2 M0/T1–T2, N2, tumours

There is no standard. The options are: surgical excision plus neck dissection, radical resection followed by postoperative radiotherapy, postoperative radiochemotherapy, external radiotherapy plus brachytherapy, hyperfractionated radiotherapy or combined radiochemotherapy. External radiotherapy should be considered if the tumour is totally exophytic. All patients should be considered for entry into controlled trials.

The macroscopic appearance of the tumour (exophytic or ulcero-infiltrating) can dictate the choice of treatment. Surgery is preferable for infiltrating lesions (level of evidence C). Radiotherapy associated with brachytherapy gives equivalent results to surgery. This is preferable to combination surgery/radiotherapy in exophytic disease or in those cases with minimal infiltration when the predicted functional outcome following surgery is important

© 2001 FNCLCC

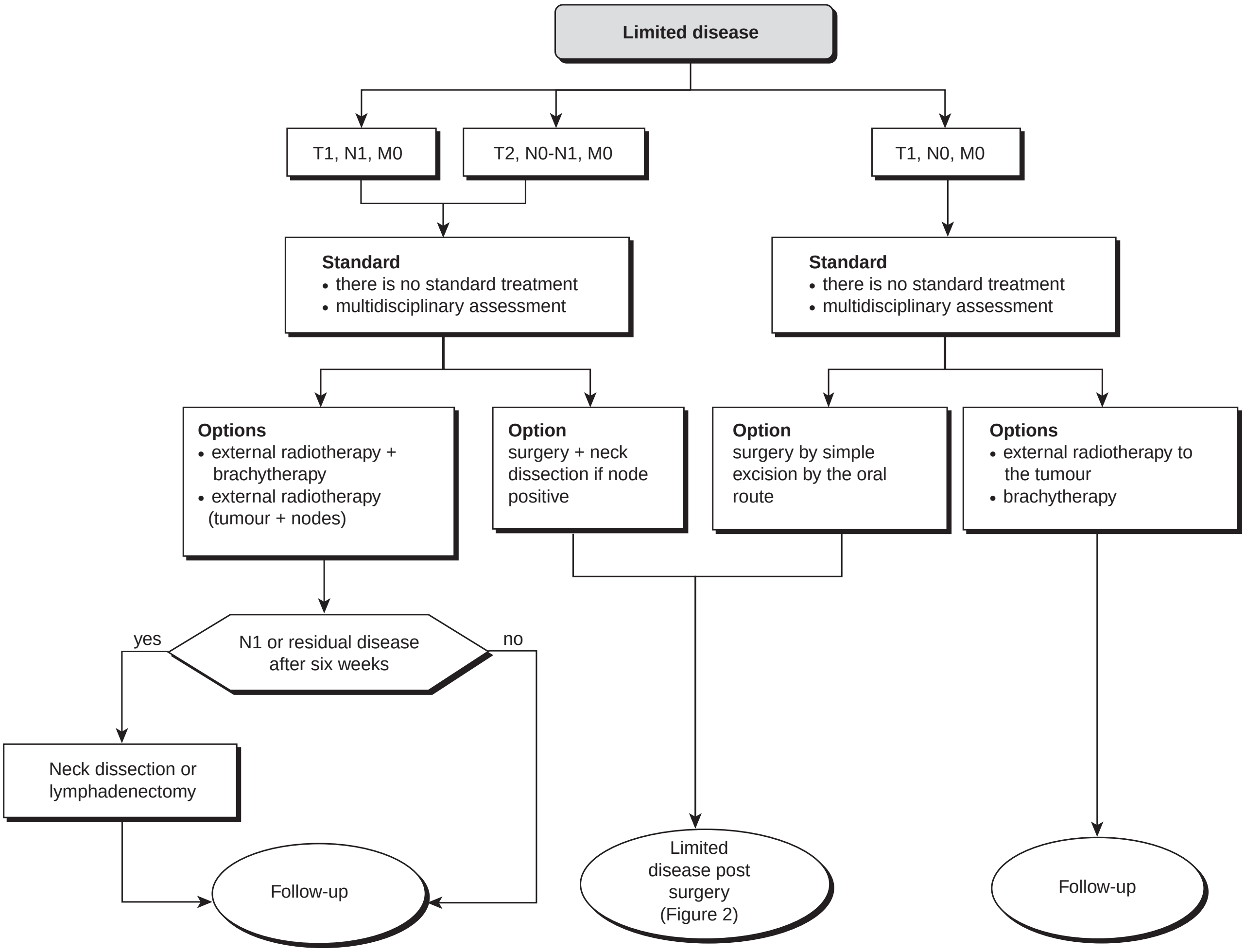

**Figure 1** Treatment of limited-stage carcinoma of the oropharynx

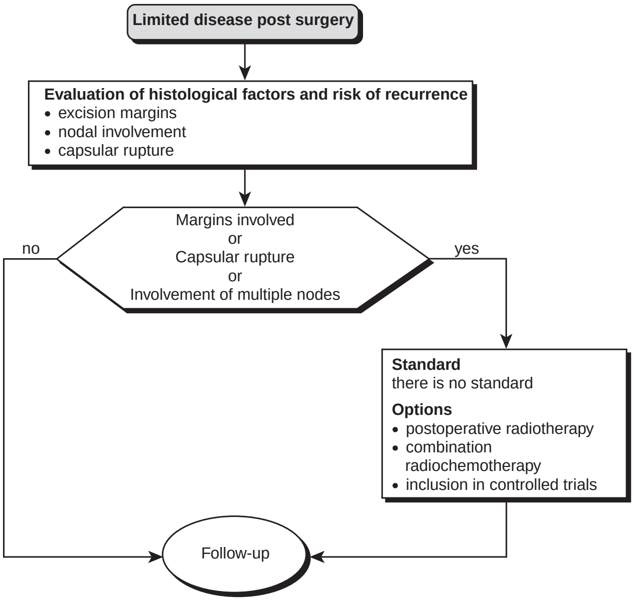

**Figure 2** Postoperative treatment of limited-stage disease

(level of evidence B). The combination of surgery and postoperative radiotherapy is more effective than radiotherapy alone, or radiotherapy associated with brachytherapy for extensive ulcero-infiltrative tumours (level of evidence B). The addition of chemotherapy either combined with radiotherapy or given postoperatively, significantly increases local control and survival (level of evidence A), but also increases morbidity. At present, there is no consensus as to the role of hyperfractionated radiotherapy.

The are various surgical methods (e.g. differences in route of approach, techniques of reconstruction, etc), but there is little difference with respect to functional result. There is no justification for the routine resection of the mandible, except when there is obvious invasion of bone. Postoperative specialist rehabilitation that includes functional aids for every-day living must be offered to patients.

In view of the frequency of microscopic nodal involvement, cervical lymph node areas should be treated routinely. Cervical clearance is always preferable to radical clearance because of the difference in functional outcome and because the rate of local control is the same (level of evidence B). For patients with N1 disease, neck dissection or adenectomy is indicated if nodes persist following potentially curable external radiotherapy. This additional surgery is generally recommended if the nodes were originally larger than 3 cm.

*© 2001 FNCLCC*

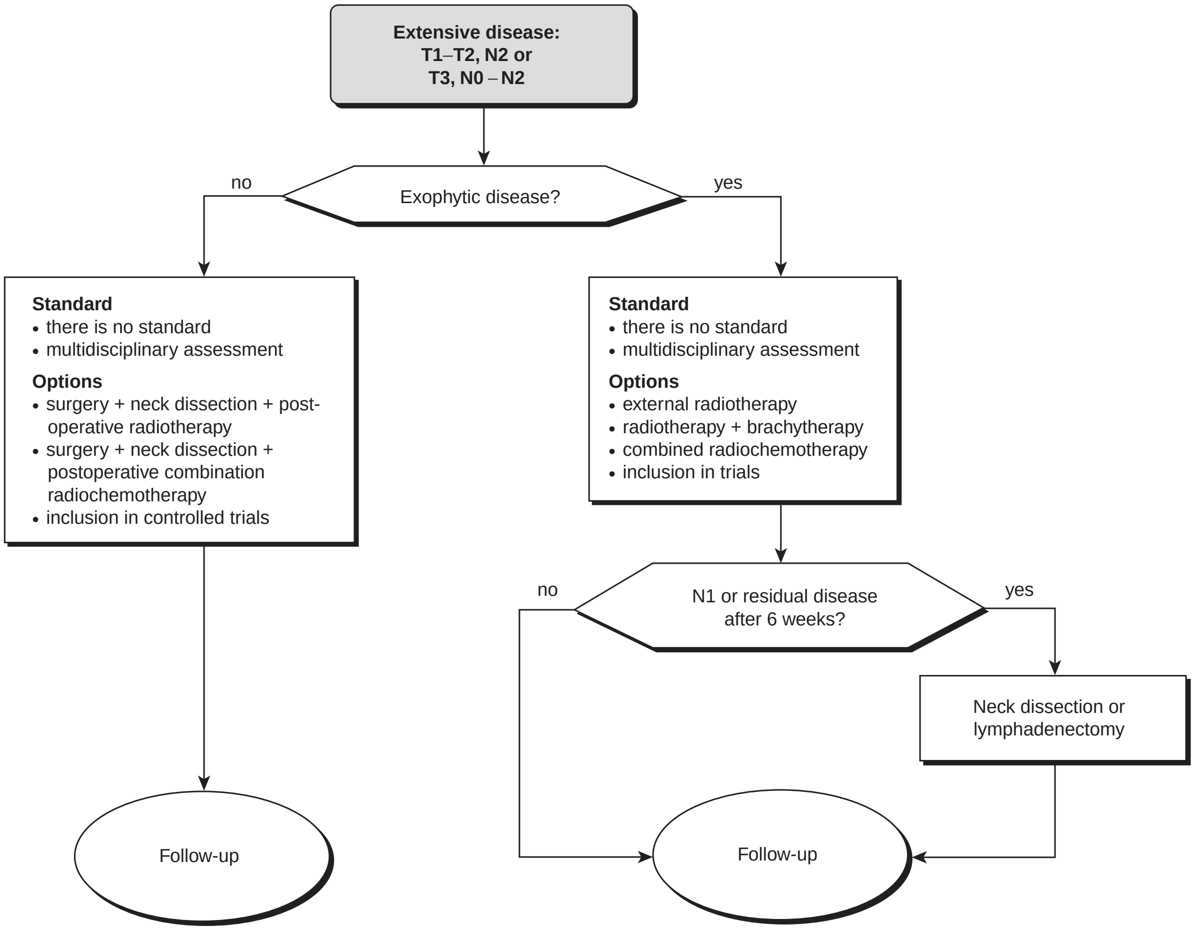

**Figure 3** Treatment of extensive disease

### T4, N0–N2, M0/all N3 tumours

There is no standard. Treatment and prognosis depends on the operability of the primary tumour and/or lymph nodes.

For stage T4, N0–N2, M0/all N3 disease with resectable tumour and nodes the options are:

- surgery plus postoperative radiotherapy
- surgery plus concomitant radiochemotherapy
- concomitant radiochemotherapy alone.

Patients should be included in therapeutic trials whenever possible.

For resectable tumours, the combination of surgery and radiotherapy is the most efficacious treatment with a control rate in the order of 60–70% (level of evidence B). Postoperative radiochemotherapy or radiochemotherapy alone are options, if possible within controlled trials. The surgical methods utilised (i.e. the route of approach and methods of reconstruction) will depend on the expertise and experience of the surgeon, who must be familiar with the diverse techniques used in these complex situations. In those patients refusing surgery, radiochemotherapy and hyperfractionated radiotherapy given within a study can be considered.

For non-resectable T4, N0–N2, M0/all N3 tumours, external radiotherapy and experimental treatment within controlled trials are therapeutic options. Combined radiochemotherapy, with radiotherapy protocols evaluating different schema of hyperfractionation, brachytherapy, new types of ionizing radiation and hyperthermia are being evaluated. The primary aim of treatment is palliation. External radiotherapy will occasionally allow subsequent surgery of curative intent. Patients should be included in controlled trials whenever possible.

### FOLLOW-UP

Clinical examination, naso-fibroscopy of the upper aerodigestive tract, and clinical assessment of nodal areas are routine investigations. An annual chest X-ray is justified in those patients at risk of a bronchial cancer. Additional investigations are undertaken according to symptomatology. In the case of suspicion of loco-regional recurrence or distant spread, the evaluation should be the same as the initial assessment.

The recommended frequency of follow-up is: clinical examination every 3 months for the first 2 years, then every 6 months for the following 3 years, then annually.

### INTERNAL REVIEWERS

JP Armand (Institut Gustave Roussy, Villejuif), A Banal (Centre René Huguenin, Saint-Cloud), C. Borel (Centre Paul Strauss, Strasbourg), S Bourdin (Centre René Gauducheau, Nantes), B

 © 2001 FNCLCC

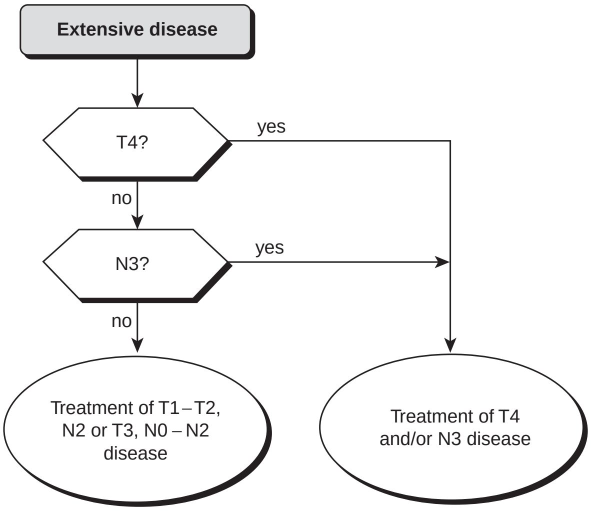

**Figure 4** Assessment of advanced-stage disease

Castelain (Centre Oscar Lambret, Lille), G Catimel (Centre Léon Bérard, Lyon), C Chenal (Centre Eugène Marquis, Rennes), N Daly-Schveitzer (Institut Claudius Régaud, Toulouse), D de Raucourt (Centre François Baclesse, Caen), L Geoffrois (Centre Alexis Vautrin, Nancy), S Helfre (Centre René Huguenin, Saint-Cloud), JC Horiot (Centre Georges-François Leclerc, Dijon), G.M Jung (Centre Paul Strauss, Strasbourg), R Le Fur (Hôpital Charles Nicolle, Rouen), TD Nguyen (Institut Jean Godinot, Reims), P Marandas (Institut Gustave Roussy, villejuif), D Peiffert (Centre Alexis Vautrin, Vandœuvre-lès-Nancy) and G Schwaab (Institut Gustave Roussy, Villejuif).

### EXTERNAL REVIEWERS

JM Ardiet (Clinique Saint-Jean, Lyon), N Barbet (Centre Claude Nuytten, Mâcon), I Bartholomot (Saint-Nazaire), P Bergerot (Saint-Nazaire), P Bertoin (Lyon), M Béthouart (Roubaix), G Boachon (Hôpital Saint-Luc & Saint-Joseph, Lyon), D Brasnu (Hôpital Laënnec, Paris), P Breton (Centre Hospitalier Lyon Sud, Pierre Bénite), D Dehesdin (CHRU Hôpital Charles Nicolle, Rouen), P Desprez (Centre Saint-Yves, Vannes), M Dray (Reims), F Economides (Dunkerque), M Finck (Clinique du Parc, Croix), D Fric (Clinique du Mail, Grenoble), D Heymans (Centre Saint-Michel, La Rochelle), D Langeron (Centre Hospitalier Fleyriat, Bourg-en-Bresse), P Manivit (Hôpital Claude Bernard, Metz), P Martin (Clinique de Louvière, Lille), JJ Mazeron (Hôpital de la Pitié Salpétrière, Paris), X Michel (Hôpital du Bon Secours, Metz), K.T Nguyen (Roubaix), C Pasteris (Clinique de Savoie, Annemasse), JJ Pessey (CHU Hôpital Rangueil, Toulouse), J.C Pignat (Hôpital de la Croix Rousse, Lyon), J Pinto (Nice), J.L Reynoard (Angoulème), G Rosan (Centre Hospitalier, Gleize), N Rouverand (Clinique de la Louvière, Lille), P Seguin (CHU Hôpital de Bellevue, Saint-Etienne), JP Suchaud (Centre Hospitalier, Roanne), JF Szterner (Hôpital Jean Monnet, Epinal), B Talon (Clinique Jeanne d'Arc, Lyon), L Traissac (Hôpital Saint-André, Bordeaux), M Vincent (Hôpital Saint-Luc & Saint-Joseph, Lyon) and M Zanaret (CHU Hôpital de la Timone, Marseille).

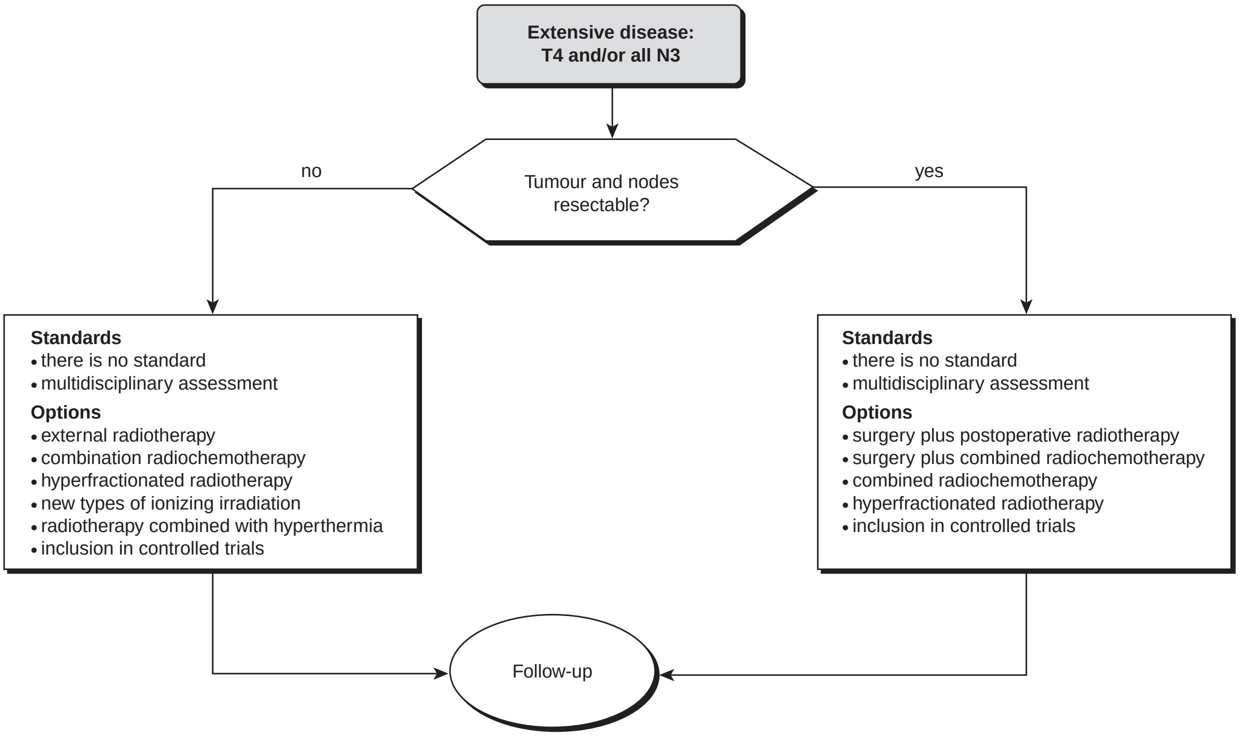

**Figure 5** Treatment of advanced-stage disease

*© 2001 FNCLCC*

